# Invasive termites in a changing climate: A global perspective

**DOI:** 10.1002/ece3.2674

**Published:** 2017-01-15

**Authors:** Grzegorz Buczkowski, Cleo Bertelsmeier

**Affiliations:** ^1^ Department of EntomologyPurdue UniversityWest LafayetteINUSA; ^2^ Purdue Climate Change Research CenterPurdue UniversityWest LafayetteINUSA; ^3^ Department of Ecology and EvolutionBiophoreUNIL‐SorgeUniversity of LausanneLausanneSwitzerland

**Keywords:** biological invasions, climate change, consensus model, global change, invasion ecology, invasive termites, species distribution models

## Abstract

Termites are ubiquitous insects in tropical, subtropical, and warm temperate regions and play an important role in ecosystems. Several termite species are also significant economic pests, mainly in urban areas where they attack human‐made structures, but also in natural forest habitats. Worldwide, approximately 28 termite species are considered invasive and have spread beyond their native ranges, often with significant economic consequences. We used predictive climate modeling to provide the first global risk assessment for 13 of the world's most invasive termites. We modeled the future distribution of 13 of the most serious invasive termite species, using two different Representative Concentration Pathways (RCPs), RCP 4.5 and RCP 8.5, and two projection years (2050 and 2070). Our results show that all but one termite species are expected to significantly increase in their global distribution, irrespective of the climatic scenario and year. The range shifts by species (shift vectors) revealed a complex pattern of distributional changes across latitudes rather than simple poleward expansion. Mapping of potential invasion hotspots in 2050 under the RCP 4.5 scenario revealed that the most suitable areas are located in the tropics. Substantial parts of all continents had suitable environmental conditions for more than four species simultaneously. Mapping of changes in the number of species revealed that areas that lose many species (e.g., parts of South America) are those that were previously very species‐rich, contrary to regions such as Europe that were overall not among the most important invasion hotspots, but that showed a great increase in the number of potential invaders. The substantial economic and ecological damage caused by invasive termites is likely to increase in response to climate change, increased urbanization, and accelerating economic globalization, acting singly or interactively.

## 1 Introduction

The spread of exotic species, climate change, and urbanization are among the most serious global environmental threats. Each factor is independently capable of effecting significant changes in biological communities, and all three have been the subject of extensive research in the context of conservation and the control of pests (e.g., Dukes & Mooney, 1999; Hudson et al., 2014; Sala et al., 2000; Walther et al., 2009). More recently, studies investigating the connectedness of these factors and their potential cumulative interactions have become more common (e.g., Brook, Sodhi, & Bradshaw, 2008; Buczkowski & Richmond, 2012; Gallardo & Aldridge, 2013; Mooney & Hobbs, 2000; Stachowicz, Terwin, Whitlatch, & Osman, 2002). Although such studies are still relatively rare, the synergy between these issues is becoming increasingly evident. For example, changing climatic conditions are expected to alter global commerce routes in the future and likely increase the introduction of exotic species into new geographic regions (Bradley, Blumenthal, Wilcove, & Ziska, 2010; Hellmann, Byers, Bierwager, & Dukes, 2008).

While the degradation of ecosystem services and biodiversity by invasive species is already a major challenge, climate change is likely to increase it. There is a general consensus that the future distribution of invasive species will likely expand with climate change (Bellard et al., 2013; Dukes & Mooney, 1999; Mooney & Hobbs, 2000; Ziska & Dukes, 2014). Previous studies have shown that changes in broad climatic conditions may influence the probability of species invasions and that such effects are likely to be diverse and context‐dependent (Bradley et al., 2010; Rahel & Olden, 2008; Walther et al., 2009). In comparison with native species, invasive species are more likely to adapt to the new climatic conditions because they are usually abundant, tolerate a broad range of climatic conditions, cover wide geographic ranges, and have highly competitive biological traits (Hellmann et al., 2008). Humans inadvertently transport a wide range of species around the globe, and although many of these inoculations presumably fail because of inhospitable climate in the recipient region (Williamson & Fitter, 1996), global warming may relax this constraint. This may especially be true for insects, which are dependent on external sources of body heat (ectotherms), and whose spread has formerly been restricted by climatic barriers.

Among insects, the highly advanced eusocial societies of ants (Hymenoptera: Formicidae) and termites (Dictyoptera: Termitidae) have been especially problematic as invaders in natural, urban, and agricultural ecosystems (reviewed in Holway, Lach, Suarez, Tsutsui, & Case, 2002; Evans, Forschler, & Grace, 2013). Previous studies have modeled the potential spread of invasive ants under climate change and demonstrated that a large amount of global landmass is climatically suitable to ant invasions (Bertelsmeier, Guenard, & Courchamp, 2013; Bertelsmeier, Luque, Hoffmann, & Courchamp, 2013, 2015). However, climate change and ant invasions were not predicted to act synergistically and the impacts on invasive ants were expected to either increase or decrease depending on the taxon (Bertelsmeier, Blight, & Courchamp, 2016). Furthermore, the ant invasion hotspots were predicted to occur mainly within biodiversity hotspots (Bertelsmeier et al., 2015), which is especially problematic for biodiversity conservation.

Despite the economic and ecological importance of invasive termites, no study has modeled their potential global distribution under climate change. Termites are cryptic social insects that play an important role in the carbon cycle and act as important ecosystem engineers in most of the world's tropical ecosystems. They contribute to the carbon cycle by feeding on a wide range of living, dead, and decaying plant matter (Bignell & Eggleton, 2000; Traniello & Leuthold, 2000), by comminution of wood and other plant residues, and by modifying soil physical properties such as texture, water infiltration rates, and nutrient contents at various spatial scales (e.g., Dangerfield, McCarthy, & Ellery, 1998). Termites are widely distributed throughout the tropical and subtropical regions of the world (Eggleton, 2000), with the highest diversity found in tropical forests where they comprise the greater part of insect biomass (Bignell & Eggleton, 2000). Despite the ecological benefits of termites, they are also significant pests causing damage to human‐built structures (Su & Scheffrahn, 1998) and tropical agriculture (Rouland‐Lefèvre, 2011). In contrast to the well‐known ecological effects of other invasive social insects such as ants (Holway et al., 2002), the ecological consequences of termite invasions remain poorly understood and most research has focused on economic consequences in urban areas.

Worldwide, the number of recognized invasive termite species has increased from 17 in 1969 to 28 today and invasive termites are increasing in both number and geographic area (Evans et al., 2013). A single recent study attempted to predict the potential habitat of *Coptotermes formosanus* and *Coptotermes gestroi* in Florida using occurrence data and climate modeling (Tonini, Divino, Lasinio, Hochmair, & Scheffrahn, 2014), but a global assessment of a wider range of invasive termite species is lacking. The goal of the current project was to provide a global risk assessment for invasive termites under scenarios of climate change using 13 of the most aggressive pest species. We model suitable area globally for these 13 invasive termite species, both currently and with predicted climate change (in 2050 and 2070). Such research is crucial for identifying areas with the highest risk of invasions and for implementing proactive management responses in the case of invasions.

## 2 Methods

### 2.1 Species distribution data

Worldwide, approximately 28 termite species are considered invasive (Evans et al., 2013) and we selected 13 to include in the global projection of termite invasion risks. These species were selected based on a number of factors. First and foremost, we selected species that are the most economically and ecologically important. For example, the Formosan subterranean termite (*C. formosanus*) and the Asian subterranean termite (*C. gestroi*) are the two most destructive termite pests in the world and are responsible for most of the $40 billion annual economic impact from termite damage (Evans et al., 2013). *Coptotermes formosanus* is on the list of the “100 of the world's worst invasive species” (Lowe, Browne, Boudjelas, & De Poorter, 2000). The eastern subterranean termite (*Reticulitermes flavipes*) is native to the eastern United States, but has spread to various parts of the world including Europe, South America, and several oceanic islands (Dronnet, Chapuisat, Vargo, Lohou, & Bagneres, 2005). It is the most common and the most economically important termite in the United States and is responsible for approximately $2 billion in damage annually (Su & Scheffrahn, 1990). Similarly, the highly destructive West Indian drywood termite (*Cryptotermes brevis*), native to coastal deserts in Peru and Chile, has invaded all continents and numerous oceanic islands is more frequently introduced into new locations than any other termite in the world (Evans et al., 2013). Second, we selected species for which occurrence data in both native and introduced ranges have been adequately described. Termite taxonomy and species identification have been problematic for a long time, and only recently molecular diagnostic tools have been used to answer questions about the sources and sinks of invasive termites (Evans et al., 2013). For example, *Reticulitermes santonensis* was considered native to France, in part because it is found in forests there. However, mitochondrial DNA sequence data have shown that *R. santonensis* is an invasive population of *R. flavipes* (Austin et al., 2005), a native of southern United States introduced into France before 1840 (Bagneres et al., 1990).

Based on the above criteria we selected 13 species: *C. formosanus*,* C. gestroi*,* C. brevis*,* Cryptotermes cynocephalus*,* Cryptotermes dudleyi*,* Cryptotermes domesticus*,* Cryptotermes havilandi*,* Incisitermes immigrans*,* Incisitermes minor*,* Mastotermes darwiniensis*,* Nasutitermes corniger*,* R. flavipes*, and *Reticulitermes grassei*. The distribution records for the 13 species were obtained from various sources including the primary literature (reviewed in Evans, 2010; Jones & Eggleton, 2011; Evans et al., 2013), the IUCN database for invasive species (IUCN SSC Invasive Species Specialist Group 2012; Jones & Eggleton, 2011), and CABI's Invasive Species Compendium (CABI 2016).

Because the models should include the full range of environmental conditions under which the species can thrive, we included occurrence points from both the native and the invaded range (following Beaumont et al., 2009; Broennimann et al., 2007; Liu, Guo, Ke, Wang, & Li, 2011). It has been shown that models calibrated on native range data alone often misrepresent the potential invasive distribution and that these errors propagate when estimating climate change impacts (Beaumont et al., 2009; Broennimann et al., 2007).

We used on average 42 occurrence points to model the species’ distribution (46 points for *C. formosanus*, 61 for *C. gestroi*, 110 for *C. brevis*, 20 for *C. cynocephalus*, 44 for *C. dudleyi*, 42 for *C. domesticus*, 38 for *C. havilandi*, 21 for *I. immigrans*, 36 for *I. minor*, 20 for *M. darwiniensis*, 40 for *N. corniger*, 40 for *R. flavipes*, and 20 for *R. grassei*). In order to make robust range predictions, it is not necessary to include every single location where the species is present, but a representative cover of all climatic conditions under which the species is known to live should be included. Our occurrence records come from all continents (except Antarctica where termites do not occur) and include tropical and temperate locations, over a wide range of latitudes. Nonetheless, we excluded species with less than 20 occurrence points (see Franklin, 2009). As all the chosen modeling methods also require absence data, we generated three sets of 1,000 randomly selected pseudo‐absences with equal weighting for presences and absences (Barbet‐Massin, Jiguet, Albert, & Thuiller, 2012).

### 2.2 Climatic predictors

To construct and project SDMs predicting the current potential distribution of the 13 termite species, we used bioclimatic variables from the Worldclim database, which represent averaged values over the period 1950–2000 (Hijmans, Cameron, Parra, Jones, & Jarvis, 2005). Previous studies on climatic niches of species and biological invasions have used these variables (Wolmarans, Robertson, & van Rensburg, 2010). Instead of simply using monthly data on temperature or rainfall, which may not have a particular significance to the organism, these variables represent derived metrics (Hijmans et al., 2005) that are known to influence species distributions (e.g., temperature of the warmest quarter) (Root, Price, & Hall, 2003). The bioclimatic variables represent annual trends (e.g., annual precipitation), limiting environmental factors (e.g., temperatures of the coldest month), and seasonality (e.g., annual range in temperature and precipitation) (Hijmans et al., 2005). The spatial resolution of the GIS layers was approximately 18.5 × 18.5 km (10 arcmin).

Termite ecophysiology is insufficiently well developed to identify individual limiting environmental factors for each species, although temperature and humidity are certainly important (Clarke, Thompson, & Sinclair, 2013). We selected three variables for each species using a three‐step procedure: (1) We tested the variable importance using the variable selection procedure in the Biomod2 package and averaged relative variable importance across all available algorithms in this package, (2) we assessed pairwise correlations among all 19 bioclimatic variables, and (3) we selected the three most important uncorrelated variables (Pearson's *r* < .75) (see Table 1 for variable selection per species and the relative contribution of the variables averaged across all models). We used GIS layers with climatic change data of future scenarios using the 5th IPCC assessment report (IPCC 2014). The WorldClim database provides projections that are downscaled to the same spatial resolution as the data for “current” conditions. Future climate scenarios are based on different geophysical hypotheses of how the Earth's climate will react to the increase in the amount of greenhouse gases. Therefore, we used a range of three different geophysical global circulation models (GCMs), which simulate the climate in response to different socioeconomic storylines: the GISS‐ES‐R model; the HadGEM2‐ES model; and the MIROC‐ESM model (IPCC 2014). To account for different socioeconomic scenarios, we used two different Representative Concentration Pathways (RCPs), which represent a midrange (RCP 4.5: +1.1–2.6°C by the year 2100) and a more pessimistic scenario (RCP 8.5: +2.6–4.8°C by the year 2100).

**Table 1:** Selected variables and their relative importance (average contribution to the models in %) per species and modeling algorithm

Variables	Bioclim code	*Cbre*	*Ccyn*	*Cdom*	*Cdud*	*Cfor*	*Cges*	*Chav*	*Iimm*	*Imin*	*Mdar*	*Ncor*	*Rfla*	*Rgra*
Annual mean temperature	Bio1					27.20			17.97					
Mean diurnal range	Bio2	33.42		26.73						10.53		29.49		
Isothermality	Bio3										24.63	49.11		
Temperature seasonality	Bio4						38.51							
Max temperature of warmest month	Bio5		23.49		11.18									
Min temperature of coldest month	Bio6		60.27		55.67									50.15
Temperature annual range	Bio7								68.39					
Mean temperature of wettest quarter	Bio8							26.62			61.87			39.46
Mean temperature of driest quarter	Bio9													
Mean temperature of warmest quarter	Bio10					35.15	30.00	23.58						
Mean temperature of coldest quarter	Bio11	60.55		27.47						51.47			43.77	
Annual precipitation	Bio12												23.54	
Precipitation of wettest month	Bio13		16.24					49.80	13.64					10.39
Precipitation of driest month	Bio14	6.03												
Precipitation seasonality	Bio15										13.50			
Precipitation of wettest quarter	Bio16			45.80	33.15							21.40		
Precipitation of driest quarter	Bio17												32.68	
Precipitation of warmest quarter	Bio18					37.65	31.48							
Precipitation of coldest quarter	Bio19									38.00				

Species abbreviations in the top row are as follows (from left to right): *Cryptotermes brevis*,* Cryptotermes cynocephalus*,* Cryptotermes domesticus*,* Cryptotermes dudleyi*,* Coptotermes formosanus*,* Coptotermes gestroi*,* Cryptotermes havilandi*,* Incisitermes immigrans*,* Incisitermes minor*,* Mastotermes darwiniensis*,* Nasutitermes corniger*,* Reticulitermes flavipes*,* Reticulitermes grassei*.

### 2.3 Species distribution modeling

We used 10 statistical and machine learning methods to model the climatic niche of the 13 termite species under current and future (2050 and 2070) climatic conditions. The models were calibrated and projected using the BIOMOD2 package v.3.3.7 (Thuiller, Lafourcade, Engler, & Araújo, 2009) and included (1) generalized linear models (GLM), (2) generalized additive models (GAM), (3) generalized boosted models (GBM), (4) classification tree analysis (CTA), (5) flexible discriminant analysis (FDA), (6) multivariate adaptive regression splines (MARS), (7) random forests (RF), maximum entropy (Maxent), (9) surface range envelopes (SRE), and (10) artificial neural networks (ANN).

To validate the models, we performed 10‐fold cross‐validation. At each run, 70% of the occurrence points are selected at random and then used to train the models and the remaining 30% of occurrence points are kept for model evaluation (Guisan & Thuiller, 2005). To test predictive performance, we used with two metrics: the area under the receiver operating characteristic curve (AUC) (Fielding & Bell, 1997) and the true skill statistic (TSS) (Allouche, Tsoar, & Kadmon, 2006).

A clear limitation of species distribution modeling is that any particular prediction is contingent on the model input data. Yet, multiple sources of uncertainty create a variety of potential outputs (Buisson, Thuiller, Casajus, Lek, & Grenouillet, 2010). Here, we base our predictions on several modeling methods, global circulation models, and socioeconomic storylines. One way to deal with this “noise” and to try to filter out a signal from these multiple forecasts is to conduct consensus forecasts (Araújo & New, 2007), which superpose individual forecasts. Here, we combined models using the ten different modeling techniques with each of the three global climate models (GCM).

As individual models can vary in their predictive accuracy, their contribution to the final consensus forecasts was weighted according to their TSS. We used only the binary predictions and not the suitability indices of the individual model outputs to create the consensus prediction because continuous outputs of different modeling methods can be probabilities or indices with different mathematical meanings (Guo & Liu, 2010). However, adding individual presence–absence predictions spatially, and scaling the value to 1, produces a suitability index that can indeed be interpreted as the probability that the grid cell presents favorable environmental conditions for the species (Araújo & New, 2007).

We generated consensus models under current climatic conditions (over 10 modeling methods), and for future climatic conditions (over 10 modeling methods and three global circulation models). For future climatic conditions, this yielded a separate consensus projection per year (2050 and 2070) and socioeconomic pathway (RCP). We also calculated the standard error of the mean between climatic scenarios in order to show the extent of variation across forecasts (Barbet‐Massin, Rome, & Muller, 2013).

### 2.4 Assessing suitable area

To assess the total suitable area for each species and the changes in suitable area with climate change, we converted the consensus projections into binary (presence–absence) predictions) using the binary transformation function in Biomod2. We stacked the binary presence–absence predictions of the 13 species in order to create “invasion hotspot” maps. We then created invasion hotspot delta maps by subtracting the current hotspot map from the future hotspot map, showing pixels that are predicted to lose or gain potential invaders. We also mapped predicted range shifts for each of the 13 species showing gained, lost, and stable habitat under future climatic conditions. To assess whether the range margins have contracted or expanded, we calculated shift vectors of the range margins in all four cardinal directions (15% of the most extreme points in either direction) and we also calculated a shift vector for the center of gravity of the species distribution. Using the s.table() function in the ade4 package, we graphically compared the sizes of the different range shift vectors, to assess whether species shift preferentially in one particular direction and whether distributional changes are predominantly expected at the range margins.

## 3 Results

**Figure 1:**
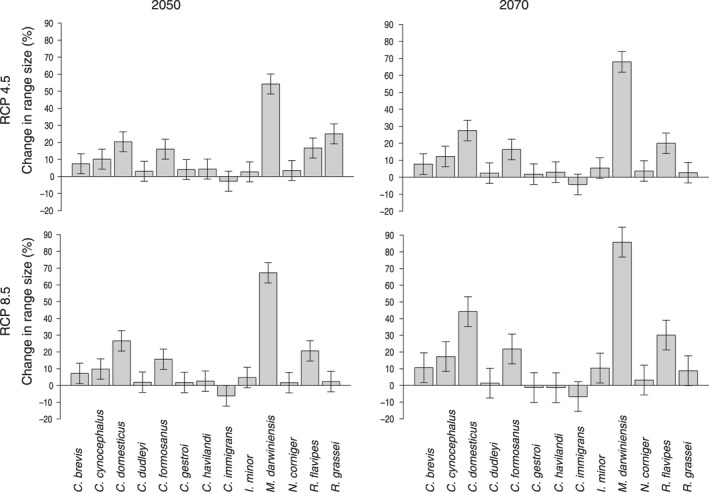
Change in potential range size (%) according to two socioeconomic storylines (RCP 4.5 and RCP 8.5) in 2050 and 2070

Most models showed fair to very good performance (Table 2), and those with insufficient TSS scores were discarded. Following climate change, almost all species (12 of 13) showed an increase in potential range size under both socioeconomic development scenarios and for both projection years. In 2050, under the RCP 4.5 scenario, all species were predicted to increase: *C. brevis* (+7.5%), *C. cynocephalus* (+10.1%), *C. domesticus* (+20.3%), *C. dudleyi* (+3%), *C. formosanus* (+16%), *C. gestroi* (+4%), *C. havilandi* (+6%), *I. minor* (+2.7%), *M. darwiniensis* (+54.2%), *N. corniger* (+3.5%), *R. flavipes* (+16.7%), *R. grassei* (25%), with the exception of *I. immigrans* which was predicted to slightly decrease (−2.8%). Under the RCP 8.5 scenario and for the year 2070, the projections were of similar magnitude (Figure 1).

**Table 2:** Evaluation metrics (AUC and true skill statistic [TSS]) for all models and species

Metric	Model	*Cbre*	*Ccyn*	*Cdom*	*Cdud*	*Cfor*	*Cges*	*Chav*	*Iimm*	*Imin*	*Mdar*	*Ncor*	*Rfla*	*Rgra*
AUC	GLM	0.944	0.972	0.968	0.93	0.965	0.942	0.919	0.894	0.887	0.952	0.953	0.901	0.99
AUC	GBM	0.942	0.966	0.959	0.932	0.95	0.953	0.931	0.862	0.901	0.959	0.97	0.926	0.904
AUC	GAM	0.94	0.731	0.947	0.908	0.945	0.917	0.918	0.646	0.897	0.798	0.985	0.87	0.824
AUC	ANN	0.932	0.977	0.954	0.942	0.949	0.945	0.918	0.793	0.893	0.913	0.929	0.859	0.977
AUC	MARS	0.953	0.828	0.962	0.885	0.988	0.939	0.909	0.663	0.862	0.97	0.959	0.861	0.981
AUC	SRE	0.775	0.9	0.947	0.908	0.793	0.858	0.913	0.62	0.73	0.783	0.845	0.868	0.903
AUC	CTA	0.898	0.95	0.897	0.892	0.876	0.881	0.893	0.808	0.773	0.867	0.913	0.89	0.887
AUC	RF	0.937	0.984	0.956	0.951	0.959	0.951	0.913	0.856	0.861	0.942	0.965	0.931	0.965
AUC	MAXENT	0.934	0.973	0.58	0.94	0.99	0.951	0.497	0.926	0.945	0.936	0.972	0.926	0.972
AUC	FDA	0.933	0.971	0.96	0.956	0.944	0.928	0.905	0.895	0.859	0.959	0.943	0.905	0.994
TSS	GLM	0.753	0.94	0.927	0.873	0.887	0.82	0.873	0.78	0.641	0.94	0.887	0.776	0.98
TSS	GBM	0.797	0.907	0.867	0.853	0.772	0.853	0.867	0.713	0.716	0.867	0.873	0.8	0.813
TSS	GAM	0.79	0.46	0.88	0.823	0.747	0.788	0.833	0.307	0.678	0.62	0.92	0.682	0.653
TSS	ANN	0.784	0.953	0.907	0.726	0.77	0.848	0.867	0.593	0.729	0.867	0.873	0.589	0.96
TSS	MARS	0.812	0.813	0.893	0.746	0.94	0.827	0.762	0.613	0.658	0.94	0.907	0.762	0.953
TSS	SRE	0.55	0.8	0.893	0.816	0.586	0.717	0.827	0.24	0.461	0.567	0.69	0.736	0.807
TSS	CTA	0.744	0.9	0.793	0.783	0.739	0.761	0.787	0.607	0.621	0.733	0.827	0.742	0.773
TSS	RF	0.782	0.967	0.893	0.86	0.777	0.833	0.776	0.687	0.742	0.813	0.9	0.833	0.84
TSS	MAXENT	0.785	0.933	0.16	0.847	0.947	0.84	0.81	0.78	0.829	0.833	0.927	0.82	0.907
TSS	FDA1	0.791	0.807	0.84	0.873	0.795	0.741	0.8	0.78	0.627	0.893	0.783	0.742	0.993

Species abbreviations as in Table 1.

**Figure 2:**
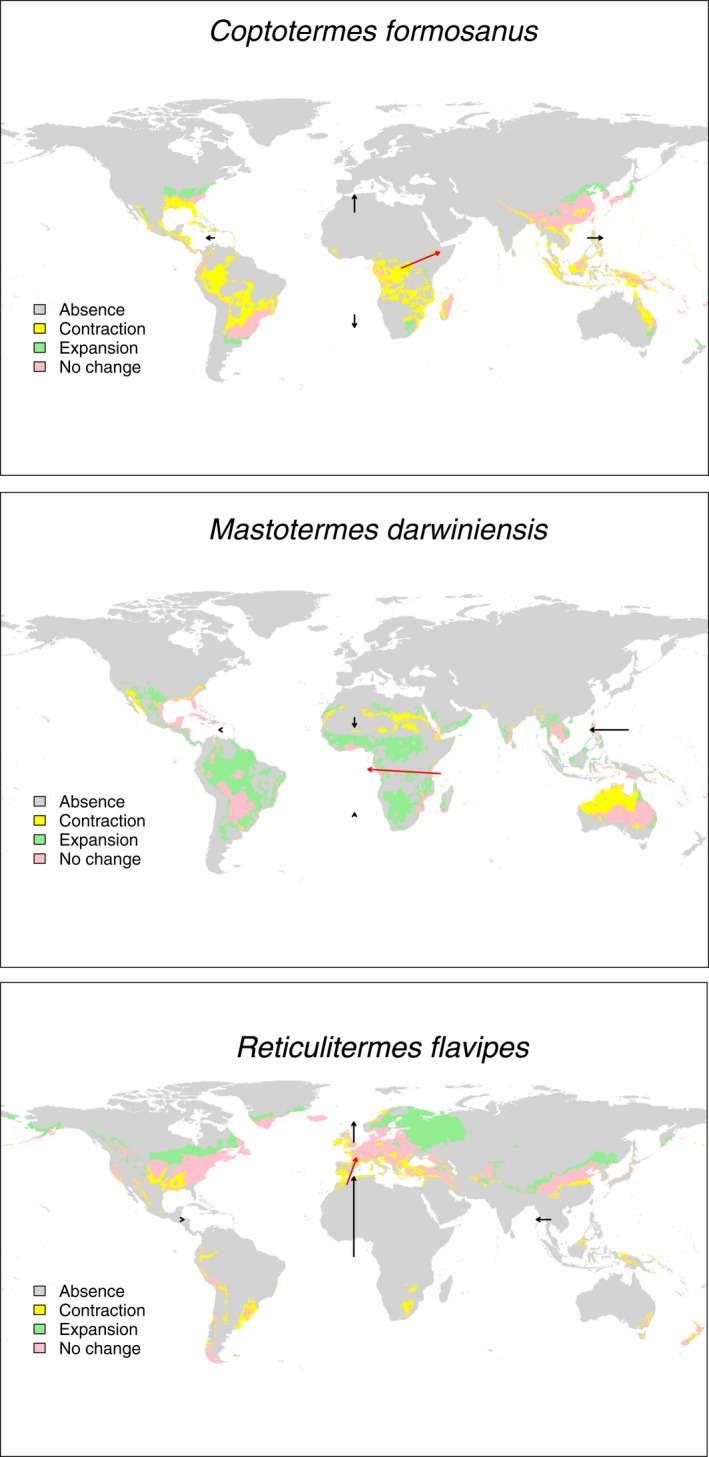
Shift maps under the RCP 4.5 2050 scenario. Areas in green are suitable in 2050 but not today (gains), areas in yellow are suitable today but not in 2050 (losses), areas in pink are suitable in both years, and areas in gray are suitable in neither of these years. The black arrows indicate changes of the range margins in all four cardinal directions, and the red arrow represents the shift vector of the center of gravity of the species potential distribution

We mapped the changes in potential ranges spatially and show the shift vectors of the range margins on the maps for each species. Given the large number of figures generated in this project (13 species × 2 time points (2050 and 2070) × 2 climate scenarios (RCP 4.5 and RCP 8.5), we only present maps for three species (*C. formosanus*,* R. flavipes*, and *M. darwiniensis*) within the main paper (Figure 2). The maps for the remaining species and scenarios are in Supporting Information. There are important species‐specific differences in spatial shifts and the areas where they are predicted to expand or contract.

**Figure 3:**
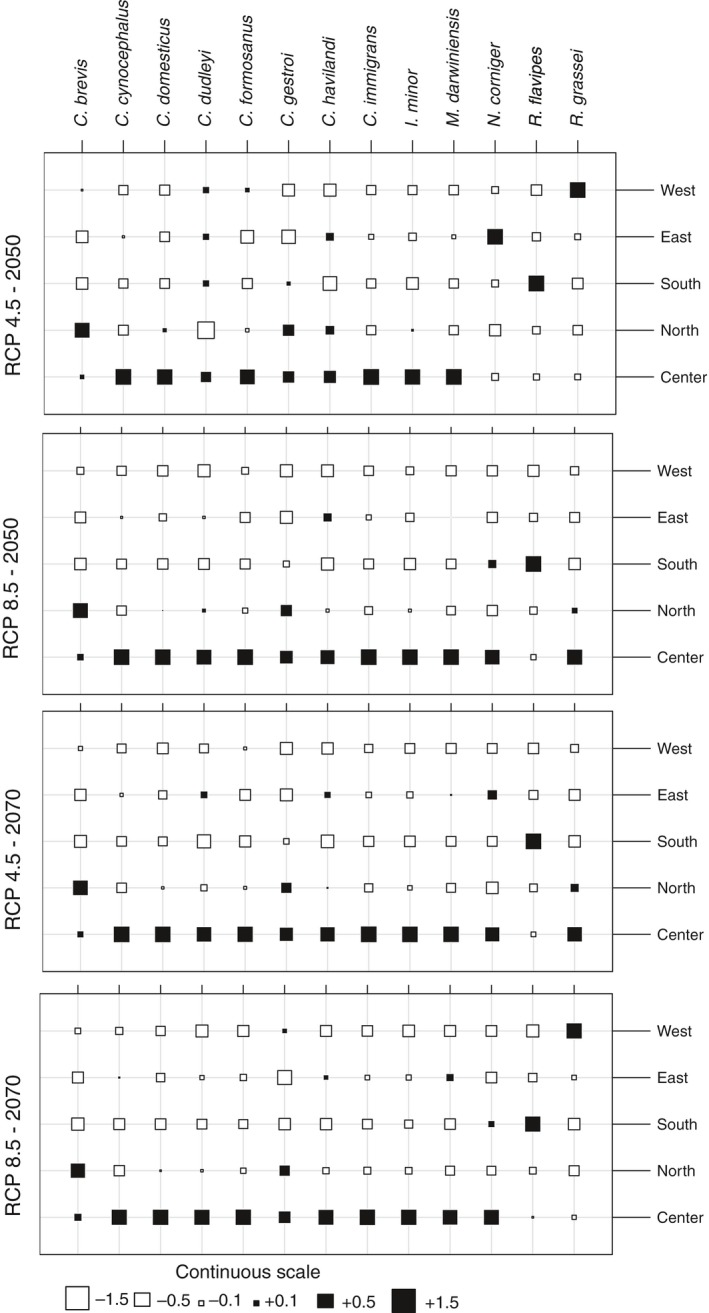
Comparison of the magnitude and direction of range shifts for 13 termite species. Range shift distance was calculated as shift vectors of the range margins and the movement of the centroid vector between the predicted distributions for baseline and future climates. Values are unitless as they are centered on the mean and divided by the standard deviation

To compare shifts at range margins and the center of gravity, we calculated the size of the shift vectors across all species and scenarios (Figure 3). The range shifts by species (shift vectors) reveal a more complex pattern of distributional changes across latitudes relative to simple poleward expansion. For most species (between 9 and 11 of 13, according to the different scenarios), the greatest changes happen at the center of the distribution and not at the range margins.

**Figure 4:**
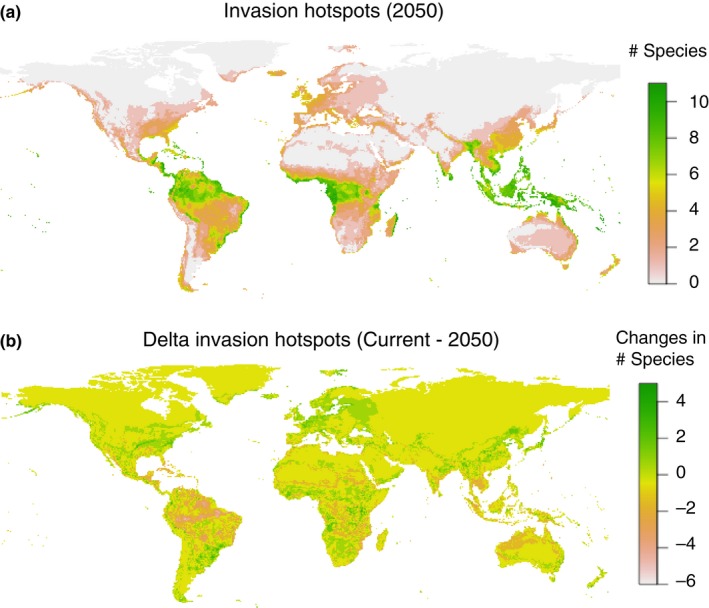
(a) Invasion hotspots in 2050 under the RCP 4.5 scenario, (b) delta invasion hotspots (number of potential invasive species per pixel in 2050—number of potential invasive species in the baseline scenario)

We mapped potential invasion hotspots in 2050 under the RCP 4.5 scenario (Figure 4a) as the number of potential invasive termite species per pixel. The most suitable areas were located in the tropics. But substantial parts of all continents had suitable environmental conditions for more than four species simultaneously (maps for RCP 8.5 and 2070 were similar to this scenario that we show here as an example and can be accessed in the Supporting Information). We also mapped the changes in the number of species per pixel (Figure 4b), revealing that areas that lose many species (e.g., parts of South America) are those that were previously very species‐rich, contrary to regions such as Europe that were overall not among the most important invasion hotspots but that showed a great increase in the number of potential invaders.

## 4 Discussion

Climate change and environmental degradation, together with increased global trade, increase the opportunities for the introduction, spread, and persistence of invasive species. Our models show that a significant global expansion is predicted for 12 of the 13 species we examined, and significant spatial shifts are observed for all species. Consequently, termite invasions will remain a global problem in urban, agricultural, and natural areas. All invasive termite species selected for the analysis belong to a relatively homogenous group and share three characteristics that together greatly increase the probability of creating viable propagules: They eat wood, nest in their food, and easily generate secondary (supplemental) reproductives (Evans et al., 2013). These characteristics combine to create propagules that may be transported outside of their native range. Such risk is especially high in lower termites (e.g., *Mastotermes*,* Reticulitermes*,* Heterotermes*) where workers are facultatively fertile and able to produce ergatoid reproductives. As a result, food resources containing foraging workers can become viable propagules any time of the year.

The joint threat posed by climate change and invasive species is growing. There is evidence that warming environments resulting from climate change are not only affecting invasive termite distribution, but also contributing to hybridization among invasive termite species. Chouvenc, Helmick, and Su (2015) reported that the two most economically important termite pests in the world, *C. formosanus* and *C. gestroi*, both invasive in Florida, are hybridizing and producing hybrid colonies with twice the growth rate of incipient conspecific colonies. Our models show that, depending on climatic scenario and projection year, *C. formosanus* is expected to increase by 15%–20% and *C. gestroi* is expected to experience slight increases of <5%. Consequently, their expansion is likely to be associated with new economic impacts and possibly novel encroachments into previously unoccupied areas, including undisturbed, native habitats.

In addition to the economic effects and damage to wooden structures, invasive termite incursions into previously unoccupied natural areas also have the potential to significantly change the ecological balance of the invaded habitats. At least eight of the 28 known invasive termite species have invaded natural habitats (Evans et al., 2013) and in many cases, the ecological consequences of such invasions have not been investigated. Such effects may be both positive (prey for other animals, positive effects on soil profiles, faster) and negative (damage to live plants, disruption of wood decomposition rates, alteration of carbon cycles, effects on population densities of native species). For example, *M. darwiniensis*, has invaded Papua New Guinea where it is infesting 42 species of native and exotic trees (Thistleton, Neal, Peki, & Dobunaba, 2007). It ringbarks and kills living trees and causes serious economic damages to various crop trees (mango, cocoa, coconut) and timber plantations. In addition, their enormous populations cause damage to infrastructure as they tunnel through various materials in the search of food. *Mastotermes darwiniensis* is considered one of the most destructive termites in the world, and our results indicate their potential range size will increase by 55%–85% globally. Similarly, *C. formosanus* in the southeastern USA has invaded native forests with significant economic and ecological consequences (Sun et al., 2007). Unlike native subterranean termites in the United States, the invasive Formosan termite infests living trees and causes damage to trees in residential landscapes, urban parks, and natural forests.

An interesting yet unexplored consequence of increased termite invasions around the globe is the complex interaction among increased termite invasions, increased methane emissions, and increased climate change. Most termite species emit substantial amounts of methane (Breznak 2000; Brune 2010), and methane is major contributor to global warming (Lashof & Ahuja 2009). Furthermore, recent reports show that under changing climate, biological invasions have a profound effect on greenhouse gas emissions (Qiu 2015). This could lead to a positive feedback loop where increases in termite invasions lead to higher methane emissions, which further drives global warming, and leads to even more termite invasions and increased methane emissions.

Another important factor in the potential distribution of invasive termites is the unexplored interaction between climate change and urbanization. Habitat degradation due to urbanization and biological invasions are the two major forces driving the erosion of biological diversity worldwide (Buczkowski & Richmond, 2012; Mack et al., 2000; McKinney, 2006; Sala et al., 2000; Vitousek, Mooney, Lubchenco, & Melillo, 2007). The two processes are often tightly linked as invasive species most often invade and thrive in disturbed habitats altered by urbanization. The disturbance created by urbanization destroys the habitat of a wide array of unique native species and often creates an attractive habitat for relatively few species able to adapt to urban conditions (Buczkowski, 2010; McKinney & Lockwood, 1999). Invasive termites typically spread with infested timbers and termites typically invade human‐modified environments before they spread to more native habitats. For example, a recent study utilized occurrence data and climate modeling to predict the potential habitat of *C. formosanus* and *C. gestroi* in Florida and demonstrated that future distribution projections for both species were influenced by urban development more than by climate change (Tonini et al., 2014). Another negative outcome of increased termite invasions is a potential increase in pesticide use in urban and natural landscapes, which could lead to broader ecological impacts on invertebrate species composition and food webs.

The known 28 invasive species are likely to increase their ranges, as 10 of the 17 known invasive species did between 1969 and today. The spatial spread of invasive termite species is a consequence of a combination of intrinsic and extrinsic factors that shape the species’ population dynamics. Intrinsic factors include dispersal, growth, survival, and reproductive constraints dictated by the species’ physiological capabilities. Extrinsic factors include factors such as the spatial and temporal availability of suitable habitat for survival, growth, and reproduction. Human‐induced environmental changes, most notably climate change and urbanization, are likely to affect both intrinsic and extrinsic factors. For example, invasive termites have been shown to adapt their reproductive phenology in response to climate change (Chouvenc et al., 2015). In parts of Florida, the dispersal flight season of *C. formosanus* and *C. gestroi* has begun to overlap due to changes in local climate. Mating pairs of heterospecific individuals were observed in the field with *C. gestroi* males preferentially engaging in mating behavior with *C. formosanus* females rather than females from their own species. This leads to hybridization between the two species and the potential evolution of highly destructive “super‐termites” due to hybrid vigor.

In summary, the substantial economic and ecological damage caused by invasive termites to is likely increase in the future as climate change, urbanization, and globalization become more pronounced and their cumulative interactions become more common. Predictive studies such as this improve our ability to pinpoint the species that are most likely to spread and the areas they are most likely to invade. Such knowledge is necessary for proactive approaches in invasive termite management including early detection and attention to high‐risk ports of entry, preventative treatments in high‐risk areas, the development of biorational IPM strategies, and public education in termite identification to effectively detect new infestations.

## Conflict of Interest

None declared.
