# Supplementary material for: Invasive termites in a changing climate: A global perspective
Source: Ecol Evol. 2017 Jan 15;7(3):974–85. doi: 10.1002/ece3.2674 (PMC5288252; doi:10.1002/ece3.2674)
Supplement: Supplementary file 1 [file ECE3-7-974-s001.pdf]

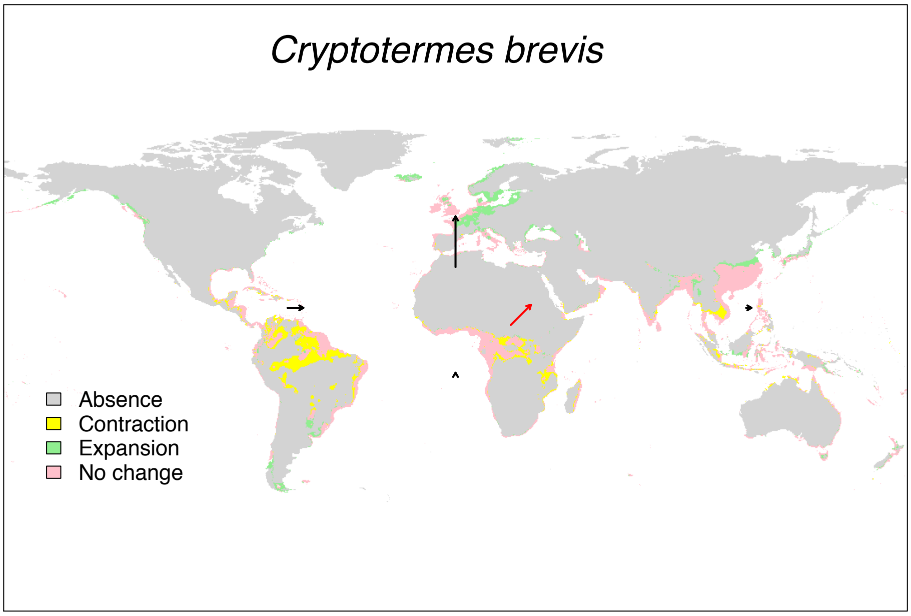
Cryptotermes brevis
Absence
Contraction
Expansion
No change

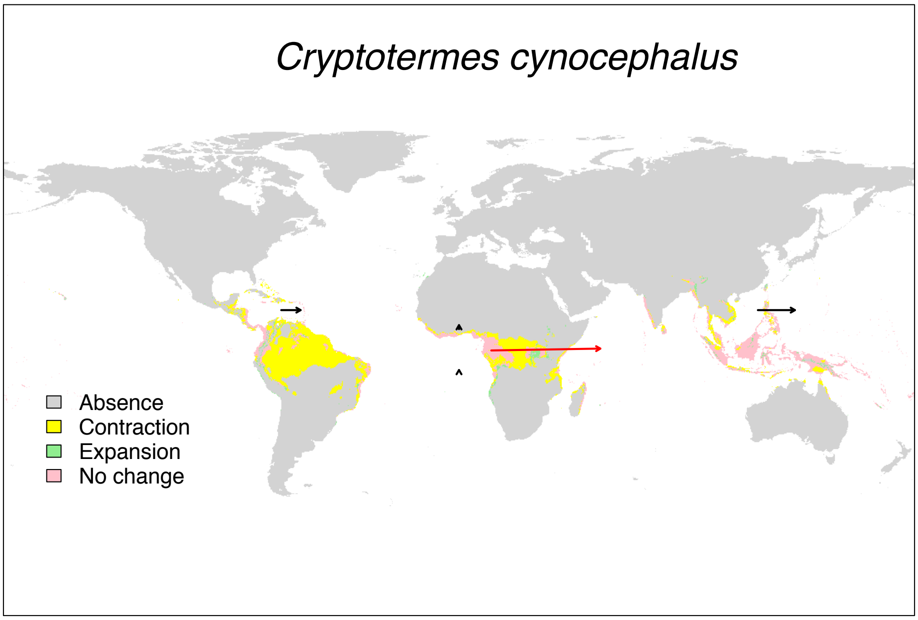
Cryptotermes cynocephalus
Absence
Contraction
Expansion
No change

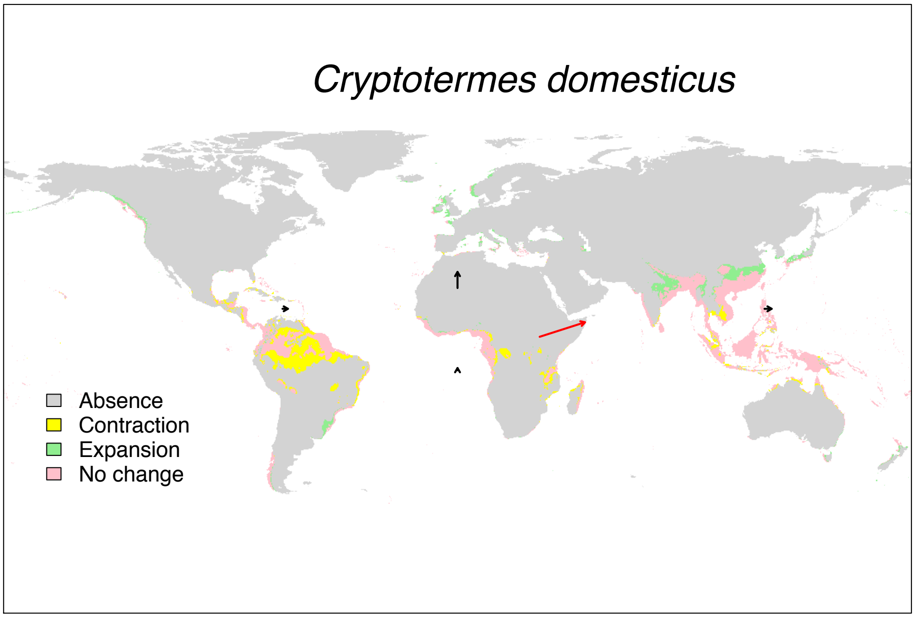
Cryptotermes domesticus
Absence
Contraction
Expansion
No change

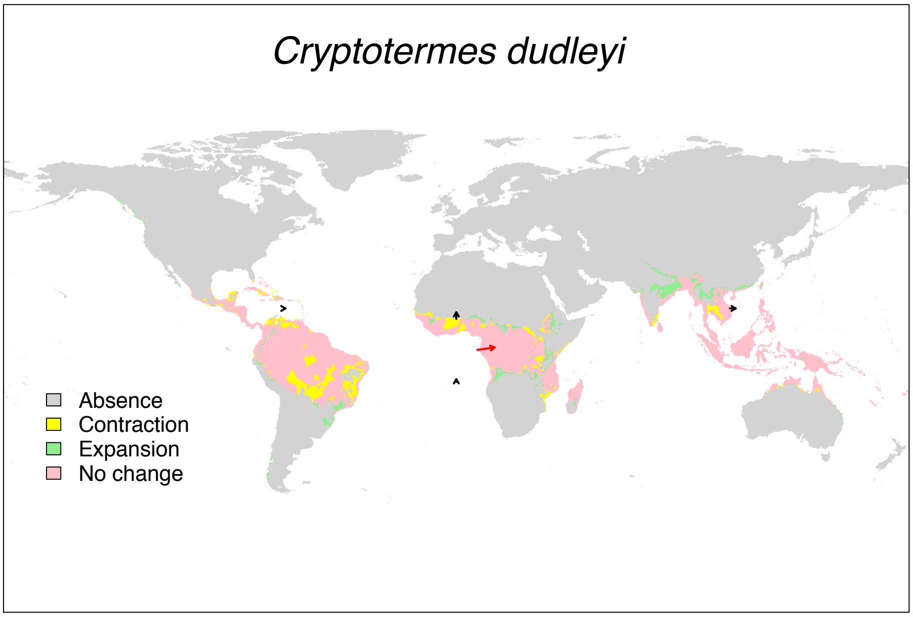
Cryptotermes dudleyi
Absence
Contraction
Expansion
No change

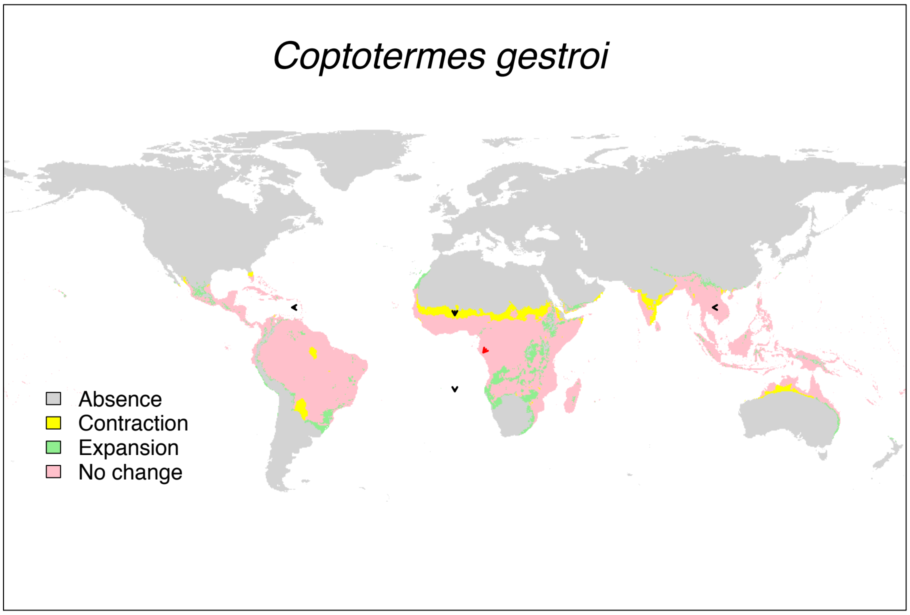
Coptotermes gestroi
Absence
Contraction
Expansion
No change

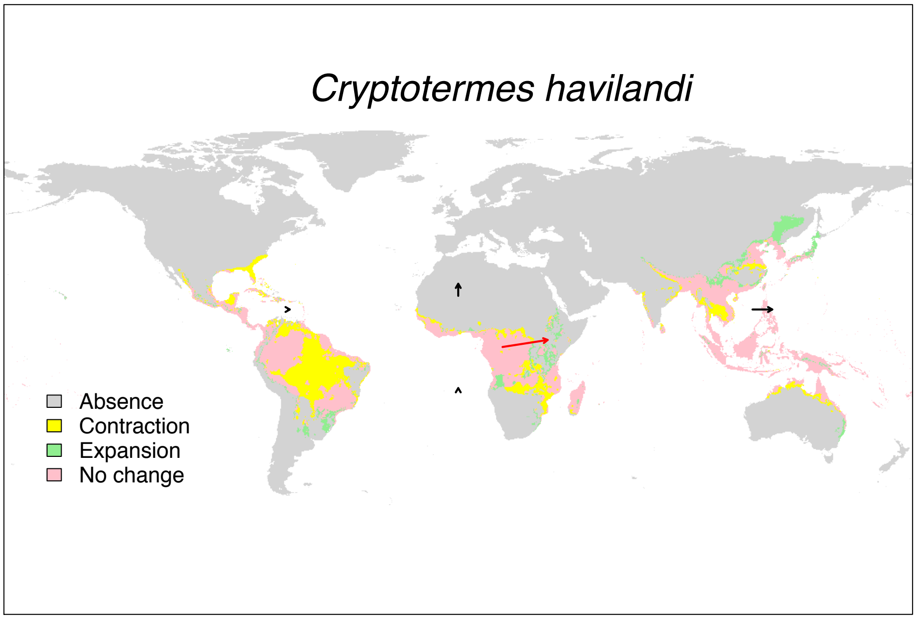
Cryptotermes havilandi
Absence
Contraction
Expansion
No change

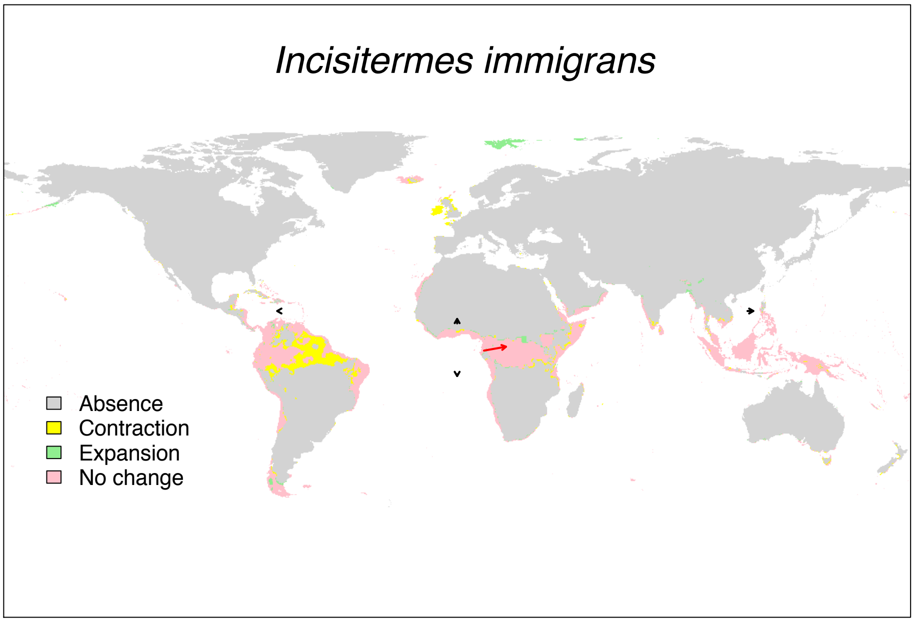
Incisitermes immigrans
Absence
Contraction
Expansion
No change

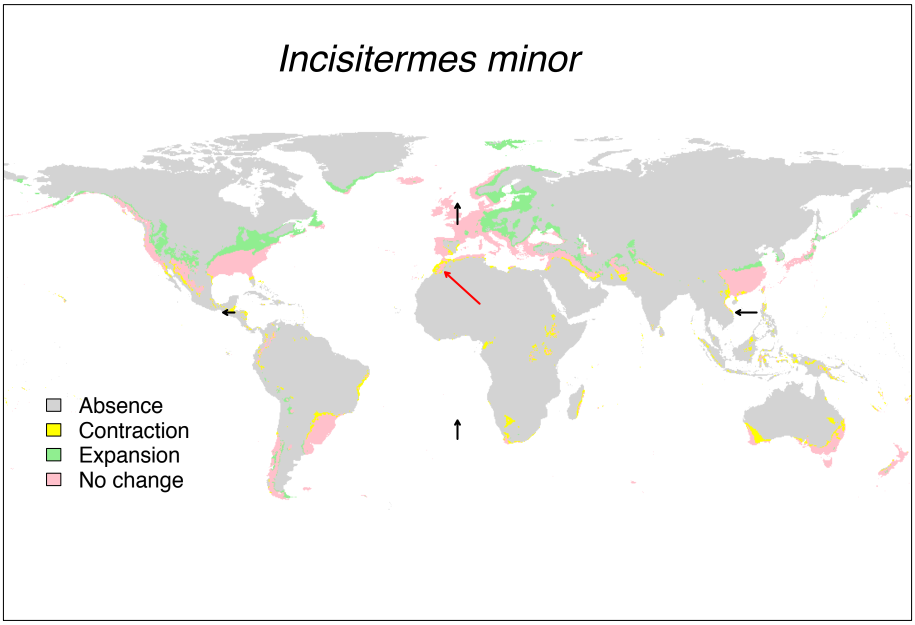
Incisitermes minor
Absence
Contraction
Expansion
No change

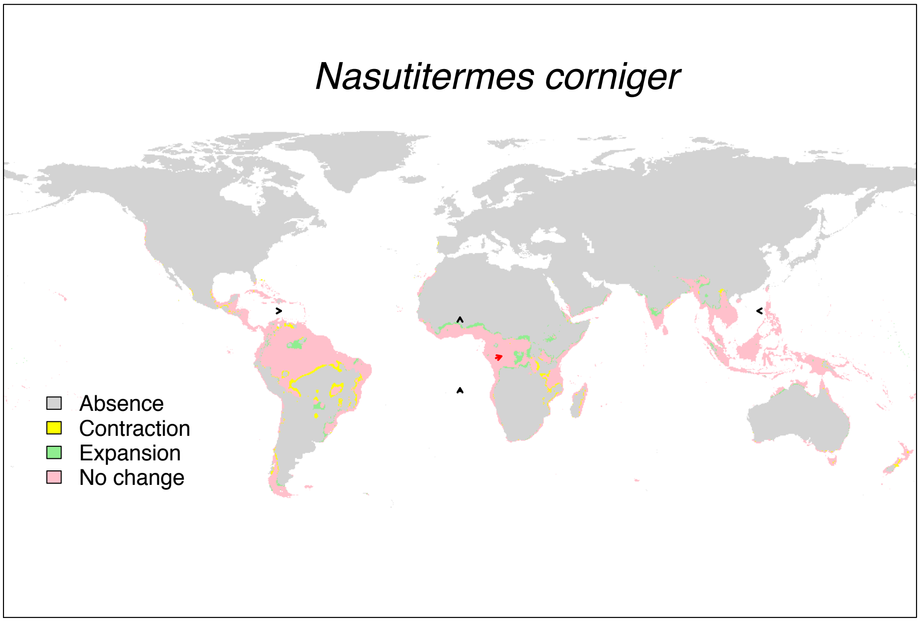
Nasutitermes corniger
Absence
Contraction
Expansion
No change

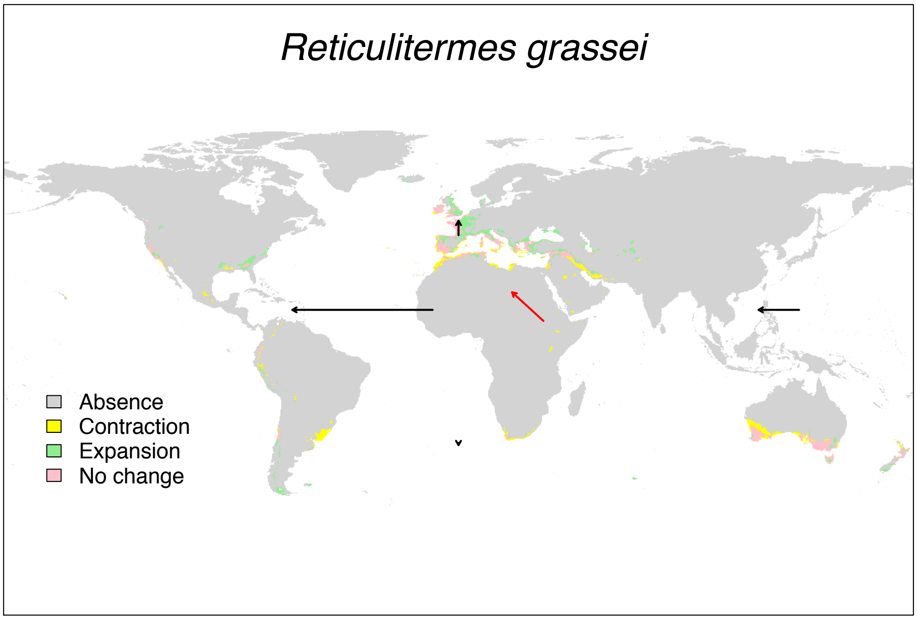
Reticulitermes grassei
Absence
Contraction
Expansion
No change
